# Deciphering the Interleukin 28B Variants That Better Predict Response to Pegylated Interferon-α and Ribavirin Therapy in HCV/HIV-1 Coinfected Patients

**DOI:** 10.1371/journal.pone.0031016

**Published:** 2012-02-06

**Authors:** Montserrat de Castellarnau, Ester Aparicio, Mariona Parera, Sandra Franco, Cristina Tural, Bonaventura Clotet, Miguel Angel Martínez

**Affiliations:** 1 Fundació irsiCaixa, Hospital Universitari Germans Trias i Pujol, Universitat Autonoma de Barcelona (UAB), Badalona, Barcelona, Spain; 2 Fundació de la Lluita contra la Sida, Hospital Universitari Germans Trias i Pujol, Badalona, Spain; Emory University School of Medicine, United States of America

## Abstract

Previous works have documented the contribution of different IL28B-associated SNPs to spontaneous HCV clearance. This study investigated the effect of different interleukin (IL) 28B genetic variants on interferon (IFN)-based therapy response. We genotyped eight IL28B single-nucleotide polymorphisms (SNPs) in a cohort of 197 hepatitis C virus (HCV)/human immunodeficiency virus type 1 (HIV-1) coinfected patients from our clinic unit who received combined pegylated (peg)-IFN-α and ribavirin (RBV) therapy. This analysis included the two strongest tag predictors for HCV clearance, rs8099917 and rs12979860, and four causal variants (rs4803219, rs28416813, rs8103142, and rs4803217) located in the IL28B promoter, coding, and 3′-untranslated regions. Haplotypes carrying the major alleles at IL28B SNPs were highly associated with sustained virological responses (SVRs) after treatment with peg-IFN-α and RBV [odds ratio (OR) = 2.5, 95% confidence interval (CI) = 1.6–4.0, 4.0×10^−5^]. Three causal SNP genotypes (rs28416813, rs8103142, and rs4803217) displayed the highest association with SVRs (OR = 3.7, 95% CI = 2.0–6.7, p = 1.3×10^−5^). All four causal variants were in high linkage disequilibrium, both among themselves (r^2^≥0.94) and with the rs12979860 variant (r^2^≥0.92). In contrast, rs8099917 was in low linkage disequilibrium with the four causal variants (r^2^≤0.45) and with the rs12979860 variant (r^2^ = 0.45). These results demonstrate that rs12979860, compared to rs8099917, may be a better predictor of response to the peg-IFN/RBV treatment among HCV/HIV-1 coinfected patients. Moreover, causal IL28B variants are strongly associated with treatment SVRs.

## Introduction

The combination of pegylated interferon alpha (peg-IFN-α) with ribavirin (RBV) has been used to treat hepatitis C virus (HCV) infection. However, a sustained virological response (SVR; a negative hepatitis C polymerase chain reaction (PCR) test 6 months after cessation of therapy) for individuals infected with HCV genotypes 1 or 4 ranges between 40% and 50%. Patients infected with HCV genotypes 2 or 3 typically achieve SVRs of nearly 75% after only 6 months of therapy [1], [2], [3], [4]. HCV genotype has been the most important predictive factor regarding the treatment response of HCV-infected patients. Nevertheless, host factors such as age, sex, race, liver fibrosis, and obesity have also been associated with peg-IFN-α/RBV therapy outcome [5], [6]. Four genome-wide association studies have demonstrated that several highly-correlated, common, single nucleotide polymorphisms (SNPs) located near the interleukin 28B gene (IL28B) strongly predict an SVR to peg-IFN-α/RBV therapy [7], [8], [9], [10]. Il28B polymorphisms have been also strongly associated with SVR in HCV/HIV-1 coinfected patients [11], [12], [13], [14], [15], [16]. Two SNPs in particular (rs12979860 and rs8099917, located 3 and 7.5 kb upstream of the IL28B gene, respectively) were the strongest predictors for HCV clearance. The recent approval of direct-acting antiviral (DAA) molecules, the NS3 protease inhibitors telaprevir and bocebrevir, active on HCV will represent a major breakthrough for HCV infected patients. Because of the low genetic resistance of first-generation protease inhibitors most failures to a triple combination of peg-IFN-α/RBV and either telaprevir o boceprevir will be due to a poor response to peg-IFN-α and RBV. Predictors of SVR to former triple combination will be also included the IL28B genotype. To determine the best SNP to predict a response to peg-IFN-α/RBV treatment, we studied the effect of different IL28B genetic variants on IFN-based therapy response. Not only will this data refine IL28b based predictions of treatment response, it may also inform studies of IL28b mechanism in HCV response.

Although the rs12979860 and rs8099917 genotypes have been independently associated with HCV treatment outcome, whether these SNPs play a causal role or are merely tagging other unknown causal variants remains to be elucidated. IL28B (which encodes IFN-λ3) up-regulates interferon-stimulated genes, similar to IFN-α and IFN-β, but via a different receptor. There is also evidence that IFN-λ 3 affects the adaptive immune response [17], [18]. Moreover, IFN-λ molecules inhibit HCV replication in vitro, and trials of IFN-λ1 in HCV-infected patients have demonstrated promising results that suggest a mechanistic link between IL28B variants and HCV treatment outcome [19]. Recently, four SNPs located in the promoter (rs4803219 and rs28416813), coding (rs8103142), and 3′-untranslated (rs4803217) regions of IL28B have been shown to be highly associated with spontaneous HCV clearance [20]. Therefore, we assessed the influence of four causal IL28B variants (rs4803219, rs28416813, rs8103142, and rs4803217) on SVR to IFN-based therapies, and compared the relationships of these four causal SNPs with the tag IL28B variants rs12979860 and rs8099917.

We previously established the strong relationships between the rs8099917 G allele and treatment failure in our cohort of HCV/HIV-1 coinfected patients [21]. In the present study, was examined the effect of eight different IL28B genetic variants on IFN-based therapeutic response in these patients.

## Results

**Figure 1:**
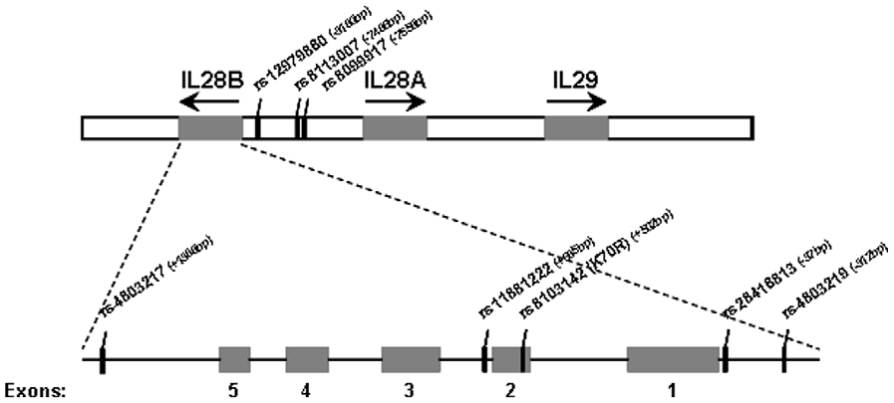
IL28B gene locus and position of single nucleotide polymorphisms (SNPs) analyzed in this study.

In a recent study, four causal SNPs (rs4803219, rs28416813, rs8103142, and rs4803217) were associated with the two tagging SNPs that were most strongly associated with spontaneous HCV clearance (rs12979860 and rs8099917) [20]. In this study, the four causal SNPs and two tagged SNPs were genotyped along with two additional SNPs, rs11881222 and rs8113007, which are located in the second IL28B intron and 7.5 kb upstream of the IL28B gene, respectively (Figure 1). These eight SNPs were genotyped in a cohort of 197 HCV/HIV-1 coinfected patients from our clinic unit who received standard combined peg-IFN-α/RBV therapy. The clinical characteristics and HCV treatment responses of the197 HCV/HIV-1 coinfected patients included in the study are summarized in Table 1. Most patients were on antiretroviral therapy (n = 196, 99.5%), and most patients had controlled HIV-1 replication (n = 169, 86%). Eighty-three patients (42%) responded successfully to HCV treatment (i.e., achieved SVR). When the HIV-1 viral load was compared in the HIV-1 viremic patients (n = 28, 14%), no significant differences were observed between SVRs and patients failing therapy (p = 0.1612, Mann-Whitney U test). Similarly, the length of time patients were under antiretroviral treatment did not impact HCV SVR (p = 0.3439, Mann-Whitney U test). HCV genotype was significantly associated with SVR in our study cohort (p = 7.2×10^−5^) (Table 1). Unexpectedly, older patients were more likely to achieve HCV SVR (p = 0.0223) (Table 1). Nevertheless, the difference between the means was very limited (48.5 vs 47 years). A moderate association with SVR was also observed with patient AST liver enzyme level (p = 0.0353), and HCV viral load (p = 0.0120). These four clinical parameters are known to be associated with HCV treatment response.

**Table 1:** Clinical characteristics of patients with chronic HIV-1 and HCV coinfection who were treated with peg-IFN-α/RBV therapy.

	SVR	NSVR	p-value
Patients, n (%)	83 (42%)	114 (58%)	-
Age (mean ± SEM)	48.57±0.5582	46.96±0.5523	0.0223
Sex, n (%)			1.0000
Female	27(44%)	36 (56%)	
Male	56 (42%)	78 (58%)	
CD4+ (cell count/ml) (mean ± SEM)	597.0±28.34	599.8±24.93	0.9866
HCV genotype, n (%)			7.2×10^−5^
1	35 (33%)	70 (67%)	
3	41 (66%)	21 (34%)	
4	11 (32%)	23 (68%)	
Other/Unknown	0	2	
ALT (U/l) (mean ± SEM)	87.78±8.22	95.19±7.30	ns
AST (U/l) (mean ± SEM)	59.31±4.95	67.88±4.04	0.0353
HCV RNA (IU/ml) (mean ± SEM)	5.819±0.079	6.043±0.048	0.0120
HCV-RNA>5×10^5^ IU/ml, n (%)	43 (49%)	83 (71%)	0.0047
Undetectable HIV-1 RNA, n (%)	68 (78%)	93 (80%)	ns

ALT: alanine aminotransferase; AST: aspartate aminotransferase; HCV: hepatitis C virus; HIV-1: human immunodeficiency virus type 1; NSVR: non-sustained virological response; SVR: sustained virological response.

Age, Mann-Whitney U test; Sex, Chi-squared test; CD4+T cell count, Mann-Whitney U test; HCV genotype Chi-squared test; ALT and AST, Mann-Whitney U test; HCV RNA, unpaired *t*-test; Undetectable HIV-1, Chi-squared test.

**Figure 2:**
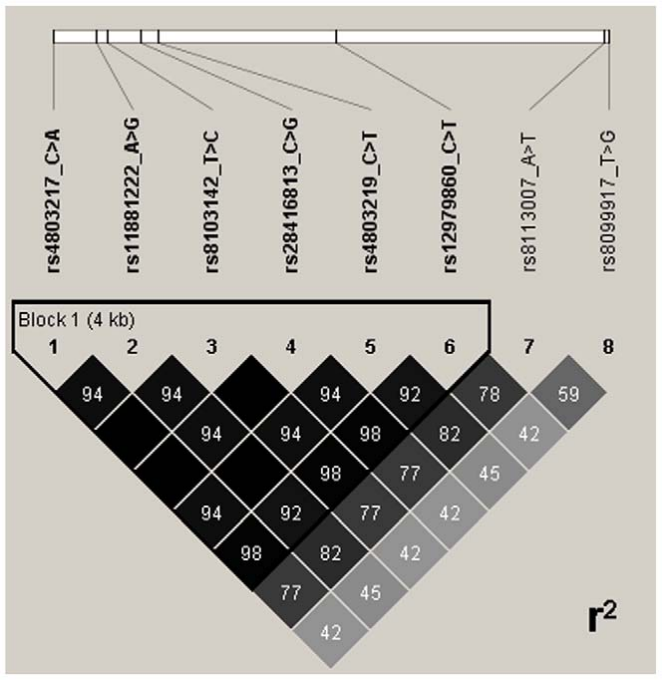
Pairwise linkage disequilibrium (LD) plot for the eight single nucleotide polymorphisms (SNPs) analyzed in this study. The linkage disequilibrium between the four candidate casual SNPs (rs4803217, rs8103142, rs28416813 and rs4803219) and the tagging SNPs is shown. An empty square represents r^2^ = 1.0.

All major IL28B single-allele SNPs were highly associated with SVRs (Table 2). Three causal major alleles, rs28416813, rs8103142, and rs4803217, demonstrated the highest individual associations with SVRs (OR = 2.9, 95% CI = 1.8–4.6, p = 6.6×10^−6^) (Table 2). As shown in Table 3, genotype distributions of all tested IL28B SNPs were significantly different among patients experiencing an SVR to peg-IFN-α/RBV treatment. Again, the causal SNPs rs28416813, rs8103142, and rs4803217 demonstrated the highest associations with SVRs (OR = 3.7, 95% CI = 2.0–6.7, p = 1.3×10^−5^) (Table 3). The four causal variants were in high linkage disequilibrium among themselves (r^2^≥0.94) and with the tag rs12979860 variant (r^2^≥0.92) (Figure 2). Importantly, rs8099917 was in low linkage disequilibrium with the four causal variants (r^2^≤0.45) and with the rs12979860 variant (r^2^ = 0.45), demonstrating that this SNP was only moderately linked to the causal SNPs in the IL28B gene. Notably, the tag rs8113007 variant (which is separated from the rs8099917 SNP by only 72 nucleotides) displayed intermediate linkage with the tag rs12979860 (r^2^ = 0.78) and the causal IL28B gene SNPs (r^2^ = 0.77–0.82). The rs11881222 SNP, located in the second IL28B intron (Figure 1), was highly linked to both the causal IL28B SNPs (r^2^≥0.92) and the tag rs12979860 variant (r^2^ = 0.92) (Figure 2).

**Table 2:** Association of individual IL28B haplotypes with sustained virological response to peg-IFN-α/RBV therapy.

SNP	Associated Allele	OR (95% CI)	p-value
rs4803217	C	2.9 (1.8–4.6)	6.7×10^−6^
rs11881222	A	2.8 (1.7–4.5)	1.6×10^−5^
rs8103142	T	2.9 (1.8–4.6)	6.7×10^−6^
rs28416813	C	2.9 (1.8–4.6)	6.7×10^−6^
rs4803219	C	2.8 (1.7–4.5)	1.6×10^−5^
rs12979860	C	2.8 (1.8–4.6)	9.9×10^−6^
rs8113007	A	2.3 (1.4–3.6)	4.0×10^−4^
rs8099917	T	2.7 (1.6–4.6)	2.0×10^−4^

**Table 3:** Association of IL28B genotype with sustained virological response to peg-IFN-α/RBV therapy.

	Allele	SVR			NSVR			MAF			
SNP	(1/2)	1/1	1/2	2/2	1/1	1/2	2/2	SVR	NSVR	OR (95% CI)	p-value
rs4803217	C/A	56	23	4	41	55	18	0.19	0.40	3.7 (2.0–6.7)	1.3×10^−5^
rs11881222	A/G	57	22	4	44	53	17	0.18	0.38	3.5 (1.9–6.3)	3.0×10^−5^
rs8103142	T/C	56	23	4	41	55	18	0.19	0.40	3.7 (2.0–6.7)	1.3×10^−5^
rs28416813	C/G	56	23	4	41	55	18	0.19	0.40	3.7 (2.0–6.7)	1.3×10^−5^
rs4803219	C/T	57	22	4	44	53	17	0.18	0.38	3.5 (1.9–6.3)	3.0×10^−5^
rs12979860	C/T	56	23	4	42	54	18	0.19	0.39	3.6 (2.0–6.5)	2.2×10^−5^
rs8113007	A/T	55	20	8	43	54	17	0.22	0.39	3.2 (1.8–5.9)	7.5×10^−5^
rs8099917	T/G	66	13	4	60	44	10	0.13	0.28	3.5 (1.8–6.8)	1.0×10^−4^

MAF: minor allele frequency; NSVR: non-sustained virological response; SVR: sustained virological response. Numbers reflect the number of patients with indicated genotype.

Haplotypes carrying major alleles at IL28B were most strongly associated with an SVR to peg-IFN-α/RBV therapy (OR = 2.5, 95% CI = 1.6–4.0, p = 4.0×10^−5^) (Table 4). These major haplotypes were visibly augmented in patients experiencing an SVR (Tables 2 and 4). Nevertheless, the prognostic value of the eight haplotypes together was not higher than the ORs obtained when the different haplotypes were analyzed individually (Table 2). Importantly, homozygosity for the three of the four causal IL28B gene SNPs, rs28416813, rs8103142, and rs4803217, was completely linked to the rs12979860 major CC genotype (r^2^ = 1.0). In contrast, the rs8099917 major TT genotype demonstrated a lower correlation with homozygosity of these three causal IL28B gene SNPs (r^2^ = 0.83).

**Table 4:** IL28B haplotype association with sustained virological response to peg-IFN-α/RBV therapy.

rs4803217	rs11881222	rs8103142	rs28416813	rs4803219	rs12979860	rs8113007	rs8099917	OR (95% CI)	p-value
**C**	**A**	**T**	**C**	**C**	**C**	**A**	**T**	2.5 (1.6–4.0)	4.0×10^−5^
A	G	C	G	T	T	T	G	0.3 (0.1–0.5)	1.1×10^−5^
A	G	C	G	T	T	T	**T**	0.9 (0.5–1.8)	0.8368
**C**	**A**	**T**	**C**	**C**	**C**	T	G	2.8 (0.7–11.4)	0.1351
A	**A**	C	G	**C**	T	**A**	**T**	0.3 (0.0–3.1)	0.3131

Major alleles are shown in bold.

## Discussion

Prior work has documented the contribution of different IL28B-associated SNPs to spontaneous HCV clearance [7], [8], [9], [10], [22]. Specifically, di Iulio et al. report the strong association of four SNPs located in the promoter, coding, and 3′-untranslated regions of IL28B with spontaneous HCV clearance [20]. However, this study did not focus on patients treated with IFN-based therapies. In the present study, we tested the influence of four causal IL28B variants (rs4803219, rs28416813, rs8103142, and rs4803217) on SVR to peg-IFN-α/RBV therapy, and compared the relationships of these four causal SNPs with the tag IL28B variants rs12979860 and rs8099917.

In the present cohort of HCV/HIV-1 coinfected patients, three major causal IL28B variant haplotypes (rs28416813, rs8103142, and rs4803217, located in the promoter, coding, and 3′-untranslated regions of IL28B) were highly associated with SVR to peg-IFN-α/RBV therapy. Moreover, these three haplotypes, which were in perfect linkage equilibrium among themselves (r^2^ = 1.0), had a higher odds ratio regarding HCV clearance than the three tag IL28B SNPs tested here (rs8099917, rs8113007, and rs12979860). Two of these tag SNPs, rs8099917 and rs12979860, demonstrated the strongest associations with HCV clearance in the above-mentioned genome-wide association studies and are currently being introduced in clinical algorithms to predict SVR to IFN-based therapies. Another important finding of our study is the strong linkage of the tag rs12979860 variant with the IL28B causal SNPs. Homozygosity for the tag rs12979860 variant completely tagged (r^2^ = 1.0) homozygosity for three major causal SNPs: rs28416813, rs8103142, and rs4803217. This result suggests that rs12979860 may be a better marker for IL28B phenotype than rs8099917 or rs8113007. These findings extend those of di Iulio et al., confirming that rs12979860 can be a better predictor for HCV clearance, as well as underscoring the strong association of causal IL28B SNPs with HCV clearance [20]. Most notably, to our knowledge this is the first study to investigate the association of IL28B tag and causal variants with patient response to peg-IFN-α/RBV therapy. These results provide compelling evidence for the relationship between IL28B tag and causal variants, and suggest possible involvement of the IL28B gene product, IFN-λ3, in SVR to IFN-based therapies. However, some limitations are worth noting. First, our study was restricted to an ethnically and epidemiologically homogeneous cohort of HCV/HIV-1 coinfected patients attending our clinic. Future work should include other ethnic and patient cohorts. Second, as previously suggested [20], functional studies are needed to precisely identify the host gene variants implicated in the control of HCV infection. Two studies found higher levels of IFN-λ2 and IFN-λ3 messenger RNAs in PBMCs from healthy individuals carrying the homozygous rs8099917 genotype associated with HCV clearance [8], [9]. However, another study observed no association between hepatic expression of IFN-λ2 and IFN-λ3 and rs8099917 genotype [23].

In short, the data shown here strongly support the association of tag and causal IL28B variants with HCV clearance after IFN-based therapy. Nevertheless, there were patients carrying alleles associated with clearance who did not clear the virus, as well as patients that carrying alleles not associated with clearance who were able to clear the virus. These results suggest that further studies should be conducted to investigate IL28B genetics, as well as other genetic factors associated with HCV clearance.

## Materials and Methods

### Patients

One hundred ninety-seven Caucasian HCV/HIV-1 coinfected patients from our HIV clinic unit who had a standard course of treatment with peg-IFN-α plus RBV with known virological response at 24 weeks post-treatment were included in this study. Treatment success (i.e., achieved SVR) was defined as undetectable plasma HCV RNA using a sensitive reverse transcriptase PCR assay 24 weeks after treatment cessation. HCV genotype, HCV viral load, HIV-1 viral load, CD4+ T cell count and liver enzyme levels.

Written informed consent was obtained from each patient who participated in the study. Similarly, ethical approval was obtained from our Institutional Review Board (Hospital Universitari Germans Trias i Pujol).

### DNA collection and extraction

Genomic DNA was extracted from peripheral blood mononuclear cells (PBMCs) using the QuickExtract DNA Extraction Protocol (EPICENTRE Biotechnologies) as previously described [21].

### Genotyping of SNPs

SNP genotyping was performed by PCR amplification and direct PCR sequencing. The rs8099917 and rs8113007 variants were PCR amplified and sequenced with oligonucleotides rs8099917–128 (5′-GTGCATATGTTTTCTGAC-3′, sense) and rs8099917–556 (5′-GAGGCCCCTCACCCATGC-3′, antisense). The PCR amplification mixture contained 5 µl of PBMC genomic DNA solution, 10 pmol of each oligonucleotide, 200 µM deoxyribonucleoside triphosphates (dNTPs), 2 mM MgSO4, 1× high-fidelity PCR buffer (Invitrogen), and 0.25 U Platinum Taq DNA polymerase (Invitrogen) in a total reaction volume of 50 µl. Cycling parameters were one cycle of denaturation at 94°C for 2 min; and 40 cycles of denaturation at 95°C for 30 s, annealing at 55°C for 30 s, and extension at 68°C for 30 s. Extension was followed by a 7-min incubation at 68°C. The resulting 430-nucleotide PCR product was sequenced using two flanking PCR oligonucleotides (rs8099917–128 and rs8099917–556) in conjunction with the Big Dye v3.1 kit and the 3100 DNA sequencing system (Applied Biosystems) as described previously [21]. Sequence alignment and editing were performed using the Sequencer version 4.1 (GeneCodes) software program. The rs12979860 SNP was amplified and sequenced with oligonucleotides rs12979860–13 (5′-GACGAGAGGGCGTTAGAGC-3′, sense) and rs12979860–595 (5′-GAGGGACCGCTACGTAAGTC-3′, antisense). PCR and sequence conditions were identical to those described above. To genotype the rs4803219, rs28416813, rs8103142, rs11881222, and rs4803217 variants, a 3308-nucleotide genomic fragment was amplified with oligonucleotides IL-28-3308F (5′-GAGCAGGTGGAATCCTCTTG-3′, sense) and IL-28-3308R (5′-AGCAGGCACCTTGAAATGTC-3′, antisense). PCR conditions were as described above, but with PCR extension for 3 min at 68°C. Sequencing oligonucleotides for this fragment were IL-28-3308F, IL-28-3308R, rs8103142RV (5′-CCATCCTCCCAGCAGTTAACCTCCC-3′, sense), 2SNPRV (5′-TCCCTCCAGCTGCTCATCTGGC-3′, sense), and rs4803217RV (5′-CCCAGACAGCCCCTGACCCA-3′, sense).

### Statistical analysis

The Mann-Whitney U test, unpaired *t*-test, and Chi-squared tests were used to analyze treatment baseline covariates. Chi-squared tests and contingency tables were used to asses host genetic associations and to calculate the p-values, odds ratios, and 95% confidence intervals. The above Statistical analyses were performed using GraphPad Prism version 4.00 for Windows (San Diego, CA, USA). Linkage disequilibrium and haplotype analyses were performed using Haploview 4.2 [24].
